# Research on the Comparison Properties of PDMS Specimens Demolding Processes and the Mechanical Performance of Hollow-Solid Ratios of Flexible Telescopic Rods

**DOI:** 10.3390/mi14061105

**Published:** 2023-05-24

**Authors:** Ruining Shang, Xiaona Li, Xiaogang Wu, Weiyi Chen

**Affiliations:** College of Biomedical Engineering, Taiyuan University of Technology, Taiyuan 030024, China; shangruining1141@link.tyut.edu.cn (R.S.);

**Keywords:** 3D-print, mold casting, PDMS, mechanics, flexible telescopic structure, transparency

## Abstract

The main motivation of this work was to demonstrate a hollow telescopic rod structure that could be used for minimally invasive surgery. The telescopic rods were fabricated using 3D printing technology to make mold flips. During fabrication, differences in biocompatibility, light transmission, and ultimate displacement were compared between telescopic rods fabricated via different processes, so as to select the appropriate process. To achieve these goals, flexible telescopic rod structures were designed and 3D-printed molds were fabricated using Fused Deposition Modeling (FDM) and Stereolithography (SLA) techniques. The results showed that the three molding processes had no impact on the doping of the PDMS specimens. However, the FDM molding process had lower surface flatness accuracy compared to SLA. The SLA mold flip fabrication exhibited superior surface accuracy and light transmission compared to the other methods. The sacrificial template method and the use of HTL direct demolding technique had no significant impact on cellular activity and biocompatibility, but the mechanical properties of the PDMS specimens were weakened after swelling recovery. The height and radius of the hollow rod were found to have a significant impact on the mechanical properties of the flexible hollow rod. The hyperelastic model was fitted appropriately with the mechanical test results, and the ultimate elongation increased with an increase in hollow–solid ratios under the uniform force.

## 1. Introduction

Telescopic structures have garnered attention in the field of biomedical engineering due to their ability to change form in response to adapting functional requirements [1]. These structures have been widely used in various industries, including aerospace [2,3], construction [4,5], and biomedical engineering [6,7]. In the medical sector, telescopic structures are frequently employed in bioprostheses [8], biomedical sensors [9], medical robots [10], and especially in minimally invasive surgery instruments. Polydimethylsiloxane (PDMS) has proven to be a versatile material for the development of medical devices and implants [11]. Its numerous advantages, such as ease of fabrication, transparency, chemical stability, plasticity, low electrical conductivity, and elasticity, have made it a popular choice in the biomedical field [12]. The properties of PDMS can be tailored to comply with specific requirements via adjusting the mixing ratios of the curing agent and diluent as well as modifying the curing conditions [13]. Previous research has primarily focused on combining PDMS with telescopic structures larger than a decimeter in size. However, considering their flexibility and potential for application in complex and narrow motion environments, PDMS-based telescopic structures on a centimeter or smaller scale could have even greater impact and significance.

The fabrication of PDMS-based telescopic structures is a serious challenge. Conventionally, PDMS-based devices have been fabricated via processes such as laser cutting, metal mold demolding, or soft lithography [14]. These techniques required a high level of technological expertise, higher production costs, and were not available for non-laboratory use. The integration of PDMS with additive manufacturing (AM) systems, specifically three-dimensional printing (3D printing), provides a promising avenue for the production of highly customized biomedical devices and implants [15,16,17]. Through utilizing silicone-based inks in combination with 3D printing technology, it is possible to achieve significant advancements in the field of biomedical engineering. To date, several PDMS 3D printing techniques have been explored, including material extrusion [18], material jetting [19], and vat photopolymerization [20]. However, these methods are not without limitations, including limited resolution and difficulties in controlling the printing of complete structures due to the high viscosity of PDMS prepolymer, as well as releasing a certain amount of ultrafine particles and volatile organic compounds during printing.

An emerging alternative is the use of 3D printing technology to make mold flips to manufacture PDMS specimens. Recently, plenty of research has been reported that using a Fused Deposition Modeling 3D printer (FDM 3D printer) to fabricate acrylonitrile butadiene styrene (ABS) and polylactic acid (PLA) molds, which were filled with PDMS. After the silicone had hardened, these molds were dissolved via organic solvent leaving the inner layer. Additionally, PLA and ABS sacrificial templates producing PDMS samples are shown to be biocompatible [21,22]. The accuracy of the 3D printing mold impacts on the light transmission of PDMS samples directly. While vapor smoothing technology has verified an ability to improve the surface flatness of FDM-printed molds [23], the associated mold size shrinkage cannot be ignored. Stereolithography (SLA) is an innovative technique that relies on selective resin curing with ultraviolet light. Compared to the FDM 3D printing process, SLA provides exceptional resolution, allowing for the creation of complex 3D molds. The light-curing resin used in SLA offers excellent light transmittance, enabling clear observation of internal PDMS bubbles, which reduces the possibility of these bubbles affecting the integrity of the PDMS specimen. Although SLA presents a wide range of possibilities for mold fabrication, the design of mold prototypes can be challenging. In addition, PDMS samples fabricated via SLA mold supported physiological cultures cells in vitro [14] and are optically transparent [24]. The available literature is limited in terms of mechanical testing of specimens produced using sacrificial template and SLA mold flip processes.

In conclusion, PDMS and flexible telescopic structures have proven to be valuable for the development of advanced medical devices and implants. The continued exploration and integration of the material with 3D printing technology offers exciting opportunities for further advancements in the field of biomedical engineering [25]. The main motivation of this work was to demonstrate a hollow telescopic rod structure that could be used for minimally invasive surgery. The telescopic rods were fabricated via using 3D printing technology to make mold flips. During fabrication, it was compared that the differences in biocompatibility, light transmission and ultimate displacement of the telescopic rods fabricated via different processes, with the object of selecting a suitable production process for mass production of telescopic rod specimens to control production costs and enhance samples’ performance. For this purpose, two advanced 3D printing methods, FDM and SLA, were applied in producing molds. Additionally, the flexible hollow rod structures were successfully fabricated for the following experiment. The biocompatibility, light transmission, ultimate elongation, and doping of the specimens fabricated via both processes were tested and compared. Simultaneously, stretch tests were performed on samples of various radii and heights of the hollow rods, and the experimental results were validated using finite element analysis (FEA) results.

## 2. Experimental and Simulation Methods

PDMS (Polydimethylsiloxane, Sylgard 184), also referred to as dimethicone, was used for molding stretch-testing samples. The material properties are mentioned in Table S1. In order to study different molding methods applied in the flexible telescopic hollow rod structure, test samples were designed. The casting samples of various radii and heights of hollow rods were tested in extension testing in order to determine the ultimate elongation.

**Figure 1:**
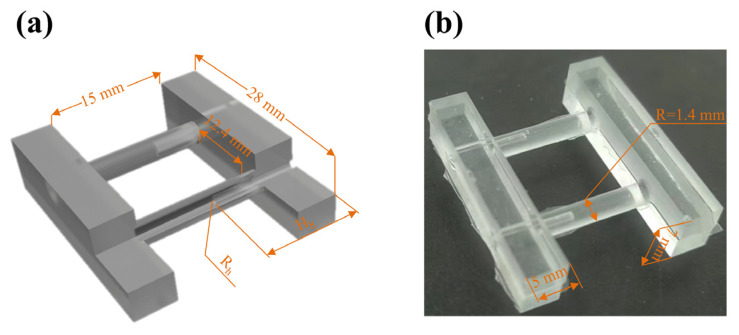
(**a**,**b**) The unit cell sizes investigated in this study.

To conform to statistical norms, three specimens were prepared for each group in this research. As shown in Figure 1, The specimen was designed to realize the expansion and contraction function. The test-piece shape was borrowed from the dumbbell-shaped test structure, due to its wider ended clamping parts in the test apparatus. Additionally, the narrower central area was tested. The design of this specimen drew on the parallel mechanism to ensure that the specimen maintained axial motion during the stretch test. Additionally, the double pipe design, as the PDMS prepolymer was filled from one side of the cavity, helped to eliminate air bubbles during the filling of PDMS due to the connected containers principle. The double pipe design also minimized the influence on the mechanical properties of the specimens due to other factors, such as manufacturing processes. The specific size of the specimens, hollow rod height (H_h_), and hollow rod radius (R_h_) are shown in Figure 1a. the model-related dimensions of which are shown in Figure 1b. Additionally, the sample H_h_ = 7.5 mm, R_h_ = 0.5 mm was regarded as the standard testing sample, which was applied in characteristic testing and the different hollow–solid ratios are shown in Table S2 (Supplementary Materials).

### 2.1. Fabrication

#### 2.1.1. Mold Fabrication

Various 3D print processes were applied in fabricating a mold, aiming to compare the differences in the performance of PDMS samples due to different demolding processes and study the mechanical effect that different hollow–solid ratios have in hollow rods. An SLA 3D printer (MicroArch S240, NanoArch, Boston, MA, USA) was used to manufacture the HTL mold (high-temperature-resistant resin) (Figure S1c). An FDM 3D printer (X1-Carbon, Bambu-Lab, Shenzhen, China) was applied in producing PLA-mold and ABS-mold (Figure S1a,b). The specimen model was independently designed by our group, and the 3D model diagram was made using SolidWorks2021 (Dassault Systems SolidWorks Corp., Waltham, MA, USA) software. The specimen was designed to realize the expansion and contraction function. The test-piece shape was borrowed from the dumbbell-shaped test structure, due to its wider ended clamp in the test apparatus. Additionally, the narrower central area was tested. The design of this specimen drew on the parallel mechanism to ensure that the specimen maintained axial motion during the stretch test. Additionally, the double pipe design, as the PDMS prepolymer was filled from one side of the cavity, helped to eliminate air bubbles during the filling of PDMS due to the connected containers principle. The double pipe design also minimized the influence on the mechanical properties of the specimens due to other factors, such as manufacturing processes. The test piece was cast from a three-part mold printed via 3D printers. For mold geometries, injection slots need to be designed to facilitate the filling PDMS. The slots were allowed the addition of excess flexible material to ensure complete filling of the mold. The over-filling could help extrude air bubbles during the casting process. A separate mold design was used to facilitate demolding, and then a scalpel was used to remove any cured overflowed PDMS in the spill area before testing.

#### 2.1.2. Fabrication Process of PDMS Samples

**Figure 2:**
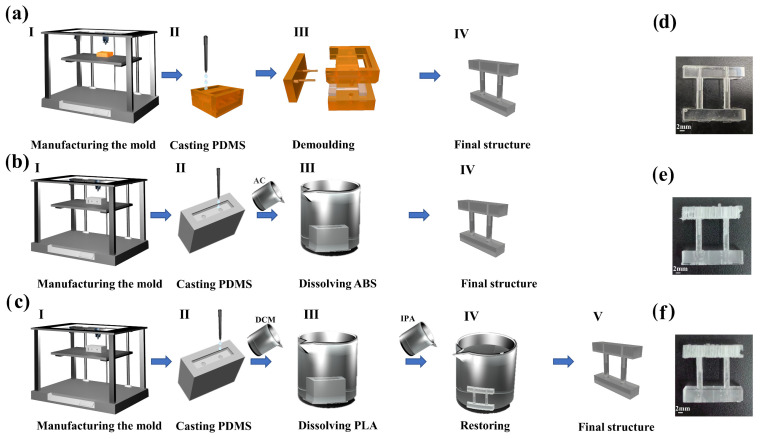
(**a**–**c**) respectively represent three types of methods for manufacturing PDMS telescopic rod structures. **a**-**I**—designing and manufacturing the mold, **a**-**II**—filling PDMS into casting mold, **a**-**III**—demolding, **a**-**IV**—cutting redundant PDMS and obtaining hollow telescopic rod. **b**-**I**–designing and manufacturing the mold, **b**-**II**–filling PDMS into casting mold, **b**-**III**–demolding, **b**-**IV**–cutting redundant PDMS and obtaining hollow telescopic rod. **c**-**I**—designing and manufacturing the mold, **c**-**II**—filling PDMS into casting mold, **c**-**III**—demolding, **c**-**IV**—restoring to normal size, **c**-**V**—cutting redundant PDMS and obtaining hollow telescopic rod. (**d**–**f**) respectively show the PDMS telescopic rod specimens manufactured by three different processes.

First, we will describe the fabrication process of PDMS test bars for the stretch-test. The process started with the creation of a hollow mold that served as a “negative part” for stretch-testing (Figure 2a-I,b-I,c-I)). The PDMS pre-polymer was then poured into the printed PLA mold and the ABS mold, penetrating the interconnected hole phases over time (Figure 2b-II,c-II). FDM printing technology allowed for both economical and straightforward fabrication of specimens, as well as hundred-micron precision and accuracy when printing thermoplastics such as PLA. In addition, the shell was designed to be continuous and thick enough to prevent leakage of PDMS into the internal areas of the mold. After curing in a 75° oven for 60 min, PLA sacrificial shells were dissolved in DCM (dichloromethane, Aladdin, Shanghai, China) solvent (Figure 2c-III) and ABS sacrificial shells were thoroughly cleaned via immersion in AC (acetone, Aladdin, Shanghai, China) solvent (Figure 2b-III), resulting in PDMS hollow telescopic rod specimens (Figure 2e,f). The PLA sacrificial template method required immersion in isopropanol solution for 6 h and drying in an oven at 60 °C for 4 h. Additionally, the ABS sacrificial template was directly placed in an oven at 60 °C for 4 h. It is aimed to allow the organic solvent between PDMS molecules to volatilize and eliminate the PDMS swelling phenomenon. HTL molds (Figure 2a-II) were made using an SLA printer, and PDMS release agent was applied to the molds. The SLA mold was then sealed with a sealing film to prevent PDMS from leaking out of the split molds. The specimens were cured in a 75° oven for 60 min and then removed from the molds (Figure 2d). These methods have proven to be effective in fabricating 3D structures with complex shapes, showing their applicability in creating complex 3D structures with hollow internal structures. Additionally, the comparison of the fabrication accuracy of all specimens has been shown in Table S2 (Supplementary Materials).

### 2.2. Stretch-Test and FEA

**Figure 3:**
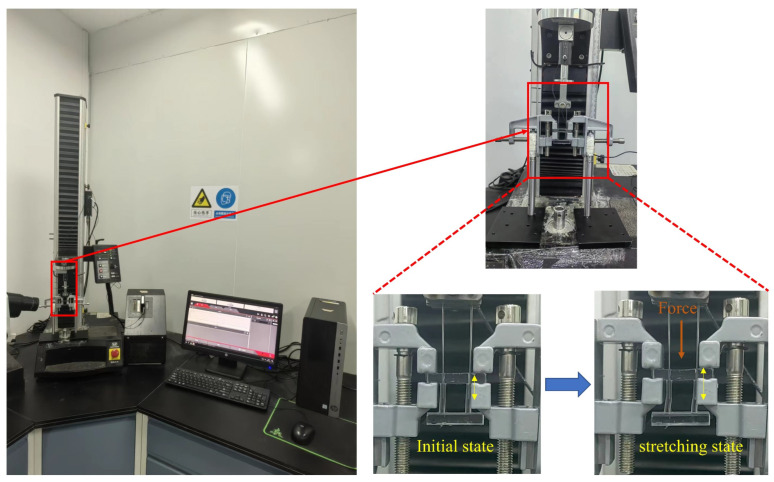
Evaluations of mechanical properties of telescopic rods were carried out on Instron 5544 tester.

The sample specimens were tested using an instron 5544 universal tensile testing machine (Instron 5544, Boston, MA, USA) with a 10.0 N full-scale load (accuracy 2%). The edges of the specimen were fixed by the clamping apparatus, leaving the middle sample to resist the loads (Figure 3). Firstly, carbide bars were soaked in silicone oil. Then, the tops of the carbide bars were clamped at the sensor chuck and the bottoms were placed at the intersection of the solid and hollow parts. Prior to the stretch-test, for each specimen, the original length L (mm) which represented the distance between grips was measured. The load from 0 to 10 N was imposed on the carbide bars at a rate of 1 mm/s. The corresponding data of load (mN) and displacement (mm) were recorded in a computer.

FEA can commonly be performed to verify experimental results. The numerical simulation is carried out using COMSOL6.0 software. Additionally, the stretch analysis of the hyperelastic structure is carried out. The hollow–solid ratios specimens with different height and different width are modeled as telescopic rod structure using the COMSOL software.

#### 2.2.1. Boundary and Initial Conditions

In this paper, a telescopic rod unit was used for the stretching experiments. On the top of the telescopic rod, the displacement boundary conditions were assumed to be 0 for transverse displacement (x = y = 0) and rotation. Additionally, longitudinal displacement (z) raised more attention. It is also supposed both lateral and rotational directions were fixed. The uniform load is applied to the hollow–solid intersection interface in the z-direction. Additionally, the value of force is set in the range of 0 to 10 N.

Furthermore, this model assumed that only the passive deformation of the unit cell is analyzed. The PARDISO direct solver and automatic height nonlinear Newton method were applied in this research. Meanwhile, the geometric multiple lattice point method was utilized for the solution.

#### 2.2.2. Material Parameters

This unit cell is made of pure PDMS. It was supposed to be an isotropic hyperelastic material. Additionally, this model was assumed to be homogeneous, isotropic, and incompressible. Additionally, the density, Poisson’s ratio, as well as Young’s modulus are showed in Table S3.

#### 2.2.3. Mesh Parameters

The FEA model for this experiment contained 42,563 tetrahedral cells. The mesh was controlled via the physical field during the mesh generation process. The meshing element type was set to tetrahedral cells. The parameters of the free mesh at the clamping part were set to coarser, and then the size of the mesh parameters of the telescopic part was set to elaborate.

### 2.3. FTIR, Transparency, and SEM

FTIR (Fourier Transform Infrared Spectroscopy) was utilized to identify the presence of particular chemical groups in the material and ascertain whether it was doped. The specimens were fabricated via three separate molding procedures and analyzed using FTIR in transmission mode. The FTIR spectra were obtained over a wavenumber range of 4000 to 550 cm^−1^, using 64 scans on a BRUKER ALPHA II GER. The FTIR spectra were normalized, and the dominant vibrational bands were linked to chemical groups.

The light transmission of PDMS samples was characterized via ultraviolet light spectrophotometer (UV-2700, Shimadzu, Kyoto, Japan). The specimens were meticulously cleaned with 75% alcohol before being placed at the colorimetric cuvette for the measurement of the translucency of the specimens. The quantification of light transmission was carried out through analyzing the specific transmission of light through each specimen. The data of transparency were recorded via the spectrophotometer’s sensor which compared light intensities from a split beam. The light source utilized provided a wavelength range of 350 to 800 nm. The initial translucency was determined through the calculation of the intensity of the monochromatic light (I_0_) and the light (I) transmitted through the specimen. The transmission coefficient (t_c_ (%)) was calculated using the equation “I/I_0_ = t_c_”. Additionally, the overall light transmission for each specimen (T) was calculated as the integration of all t_c_ values (t_c_ [λ] dλ [10^−5^]) [26]. Finally, the T value of each material was divided via the T value obtained with no specimen in the spectrophotometer to obtain the light transmission as a percentage.

In order to study the microscopic characteristics of the samples’ surface, scanning electron microscope (SEM) images were captured. At first, a thin layer of gold (10 nm) was sputtered on the sample surface. Then, images were acquired via SEM (JSM-7100F, Tokyo, Japan) at 10 kV.

### 2.4. Biocompatibility Assessment

Osteoblasts MC3T3 were used as model cells to evaluate the biocompatibility of PDMS specimens made via different demolding processes. The telescopic rod specimens were sterilized in an autoclave and then placed in fresh 24-well culture plates. Cell suspensions with a density of 10,000/well were gently dripped around and on the surface of the specimens. They were incubated for 1 day and the extract was transferred to a fresh 96-well culture plate. Fifty μL of CCK8 (Cell Counting Kit-8, Macklin, Shanghai, China) stain and five mL of medium was added to each well and incubated for 4 h. Then, the medium was discarded and 50 μL of liquid was removed and transferred to a new 96-well plate, and the absorbance at 450 nm wavelength was measured using an enzyme marker. To delve deeper into cellular activity, cell staining and live-dead experiments were conducted. The sterilized specimens were incubated with cells for one day and then subjected to live-dead (CCK8) stain. The well-plates were placed in an incubator for 30 min, protected from light, and then removed. DAPI (cell nuclei staining) was performed through adding 3.7% caprolactone to the leaching solution for 10 min, followed by 0.1% tretinoin for 5 min. After that, the DAPI staining solution was incubated for 5 min and imaged using an inverted fluorescence microscope (Ti-S, Nikon, Tokyo, Japan). The viability of the cells was determined as the ratio of live cells to the total number of cells.

## 3. Results and Discussion

**Figure 4:**
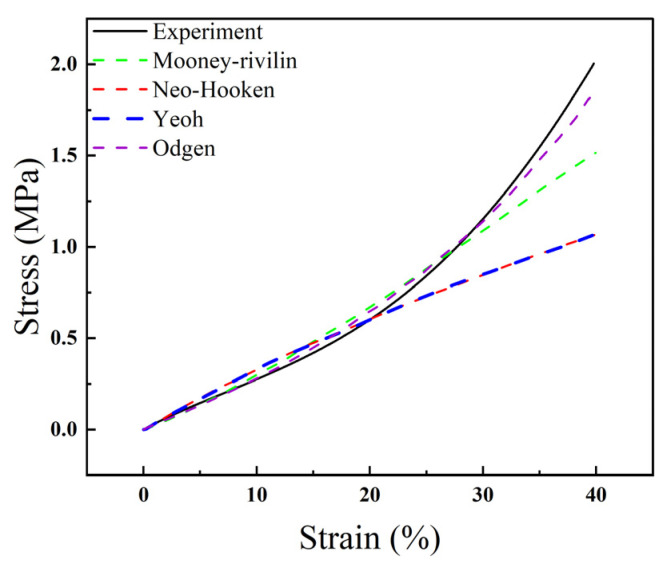
Fitted image of PDMS hyperelastic simulation for uniaxial tensile experiment.

Hyperelastic constitutive models are mathematical models used to simulate behaviors of rubber-like materials, such as silicone rubber. The commonly used hyperelasticity models are Mooney–Rivlin [27], neo-Hookean [28], Yeoh [29], and Ogden [30]. The application ranges of these hyperelastic models are shown as follows: The neo-Hookean model is often used to predict the nonlinear stress–strain behavior of rubber-like materials under large deformations. The Mooney–Rivlin model evolved from the Neo-Hookean model, and the Mooney–Rivlin model is often used to model the nonlinear behavior of incompressible materials under large strains. The Yeoh model is often used to express models for the deformation of almost incompressible nonlinear elastic materials. The Ogden model is a commonly used hyperelastic model. To enhance the accuracy of the FEA, the dumbbell-shaped standard parts were tested in tension, and fitted with the first-order hyperplastic models, such as Mooney–Rivlin, Neo-Hookean, Yeoh, and Ogden. The Ogden model fit best; thus, the first-order Ogden model was used for the FEA (Figure 4).

The first-order Ogden formula can be used to theoretically predict the deformation of the telescopic rod of modulus of *λ*_1_, *λ*_2_, *λ*_3_. Therefore, the strain energy function *U* can be simplified as [31]:

(1) $$U\left(\lambda_{1},\lambda_{2},\lambda_{3}\right)=\sum_{r=1}^{N}\frac{\mu_{r}}{\alpha_{r}}\left(\lambda_{1}^{\alpha_{r}}+\lambda_{2}^{\alpha_{r}}+\lambda_{3}^{\alpha_{r}}-3\right),$$

where *μ_r_* and *α_r_* refer to material parameters.

The Cauchy principal value stresses for a hyperplastic incompressible material as follows:

(2) $$\sigma_{i}=\lambda_{i}\frac{\partial U}{\partial \lambda_{i}}-p\;\left(i=1,2,3\right),$$

where *p* refers to an arbitrary hydrostatic pressure.

Then, a constitutive relationship is found via evaluating (1) with (2).

(3) $$U\left(\lambda_{1},\lambda_{2},\lambda_{3}\right)=\sum_{r=1}^{N}\frac{\mu_{r}}{\alpha_{r}}\left(\lambda_{1}^{\alpha_{r}}+\lambda_{2}^{\alpha_{r}}+\lambda_{3}^{\alpha_{r}}-3\right),$$

The stretching load–displacement curves and the numerically calculated homogenized stretching response are plotted together in Figure S2 at different H_h_ and R_h_ values (Supplementary Materials). The consistency between the experimental data and simulation is evident in the range of elastic deformation, confirming the validity of the numerical calculation. There is still a slight deviation between the experimental data and the numerical simulation due to fabricating-process-related factors, for example, the molding process. The parameters of FEA have been shown in Table S3 (Supplementary Materials).

**Figure 5:**
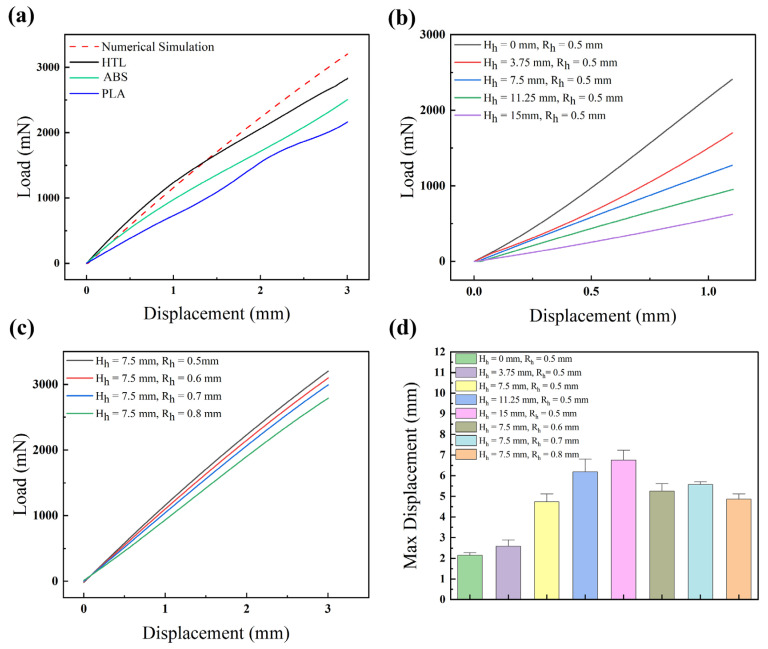
(**a**) Experimental load–displacement of PDMS specimens manufactured via different turn-flipped molding processes. (**b**) Load–displacement FEA curves of PDMS specimens with different H_h_ at the same R_h_ within the displacement (0–1 mm). (**c**) Load–displacement FEA curves of PDMS specimens with different R_h_ at the same H_h_ within the displacement (0–3 mm). (**d**) Comparison of the ultimate elongation of PDMS specimens with various hollow–solid ratios.

The results indicated that the mechanical properties of specimens produced through various manufacturing processes exhibit variability, as shown in Figure 5a. The relationship between the PDMS specimens fabricated via different processes and the theoretical calculated numerical fit is HTL > PLA > ABS. This result could be explained as follows: the application of organic solvents in the sacrificial template method for the fabrication of PDMS specimens is known to result in swelling effects. Despite having comparable solubility parameters, methylene chloride has been observed to induce a greater degree of swelling in PDMS compared to acetone [32]. The swelling phenomenon led to alterations in the surface properties of PDMS, which were believed to contribute to changes in its mechanical properties [33,34]. The results presented in Figure 5b,c and Figure S3a,b indicate that as the hollow–solid ratios increased, the displacement produced by the structure under the same thrust load increased. Furthermore, during stretch testing, the fracture surface often propagated from the contact surface between the push rod and the specimens. When the pressure applied on the contact surface, the solid state got compressed. By contrast, the hollow part presented stretching state. Figure S4 showed that stress concentration formed on the contact surface of the pull-poles and PDMS specimen. It provided an explanation for the above phenomenon.

The results depicted in Figure 5d indicate a gradual increase in ultimate elongation with an increasing percentage of hollow in the axial direction. Meanwhile, in the radial direction, ultimate elongation increases with a growing portion of the cavity. However, when the R_h_ reaches 0.8 mm, the ultimate elongation decreases. The decrease is attributed to the thinning of the tube wall, resulting in decreasing tensile strength and the attainment of fracture critical value. Additionally, variations in ultimate elongation can also be seen between specimens manufactured using different processes. The samples fabricated via SLA-3D printing of the HTL mold displayed the minimum ultimate elongation. The specimens manufactured via sacrifice mold with FDM-3D-printed ABS molds were better than the samples fabricated via SLA-3D printing of the HTL. Samples manufactured with FDM-3D-printed PLA molds showed the max ultimate elongation, as shown in Figure S3c.

**Figure 6:**
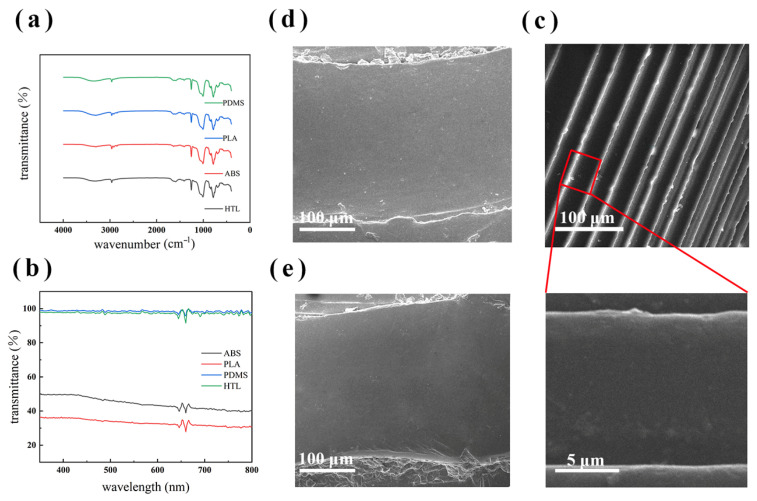
(**a**) FT-IR spectra and (**b**) UV-vis spectra of PDMS hollow telescopic rod structure made via four types of process of manufacturing. (**c**) SEM of sample made of split casting mold which made via Stereolithography (SLA) 3D printing. (**d**) SEM of samples fabricated using 3D-printed sacrificial templates (PLA) as well as (**e**) SEM of sample fabricated using 3D-printed sacrificial templates (ABS).

Figure 6a shows the FTIR spectra of pure PDMS, PDMS fabricated via ABS sacrificial mold, PDMS fabricated via PLA sacrificial mold, as well as fabricated via HTL dissected mold, respectively. The absence of the new peak proved specimens made via different processes without the presence of impurity chemical groups. As shown in Figure 6b, the relative transmittance values at 370 nm were 98.6%, 97.8%, 49.6%, and 36% for pure PDMS and PDMS produced via different processes, viz., fabricated via the HTL mold split injection molding process, ABS sacrificial template process, and PLA sacrificial template process, respectively. It was demonstrated that the light transmission of PDMS specimens made with the HTL mold flip is closer to that of pure PDMS. Comparing the SEM (Figure 6c–e) of PDMS made via different processes, it was concluded that without adulteration, the swelling phenomenon of PDMS in organic solvents and the lower precision of 3D- printing increased the roughness of the surface of samples, which led to a scattering effect, and then triggered a decrease in transparency [35].

While the processes of sacrifice mold were proved to be biocompatible and applied in cell scaffolds, Comparison of the biocompatibility of various processes has not been reported. The purpose of this study was to evaluate the potential of PDMS specimens fabricated via different processing processes for interventional instruments. Additionally, specimens with R_h_ = 0.5 mm and H_h_ = 7.5 mm were selected for studying cell viability and metabolic activity.

**Figure 7:**
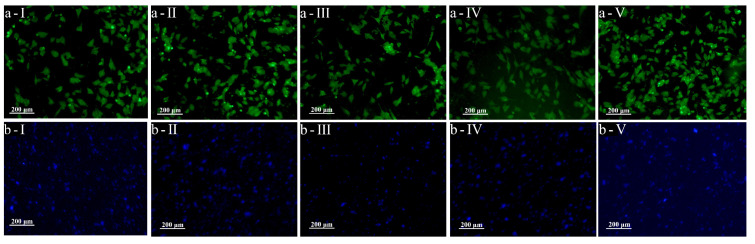
**a-I**—live-dead assay of control, **a-II**—live-dead assay of pure PDMS, **a-III**—live-dead assay of PDMS made from ABS sacrificial mold, **a-IV**—live-dead assay of PDMS made from PLA sacrificial mold, **a-V**—live-dead assay of PDMS made via HTL dissected mold. **b-I**—nucleus assay of control, **b-II**—nucleus assay of pure PDMS, **b-III**—nucleus assay of PDMS made from ABS sacrificial mold, **b-IV**—nucleus assay of PDMS made from PLA sacrificial mold, **b-V**—nucleus assay of PDMS made via HTL dissected mold.

**Figure 8:**
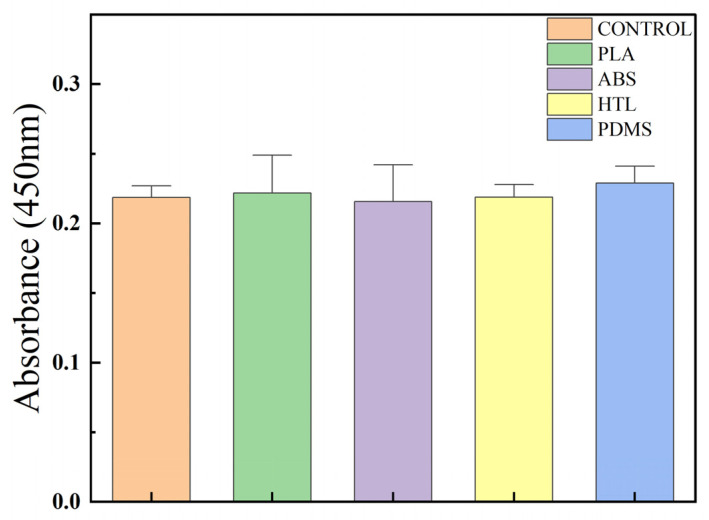
CCK8 Cytotoxicity assay of control, pure PDMS, PDMS made from ABS sacrificial mold, PDMS made from PLA sacrificial mold, and PDMS made via HTL dissected mold, respectively.

The nuclei of DAPI-stained cells showed blue fluorescence in the leaching solution (Figure 7b). It was indicated that the cells were widely distributed and with sound intercellular interactions, which was beneficial to maintaining cell viability. Live cells stained with conventional live/dead detection reagents showed green fluorescence in the leaching solution (Figure 7a). Additionally, the cells were present as cell clusters and had a shuttle-shaped morphology. The result indicated that all the PDMS specimens fabricated via the three different processes provide a good cellular environment. The results of the CCK8 test (Figure 8) showed that the OD values of the cells cultured in control, PLA, ABS, HTL, and pure PDMS leaching solution for 1 day were between 0.22 and 0.23. It is clearly demonstrated that the biocompatibility of the PDMS specimens made via three processes was not significantly different from that of the blank control and pure PDMS. Additionally, these samples maintained high cell viability. Therefore, PDMS specimens fabricated via three processes can be applied in processing interventional devices with good biocompatibility.

## 4. Conclusions

In this work, we propose a hollow telescopic rod structure that could be used for minimally invasive surgery. In order to control the cost and improve the performance of the samples in high-volume production, it is important to select a suitable production process. To this end, we compared the differences in biocompatibility, light transmission, and ultimate displacement of telescopic rods fabricated via different processes. This study showed that the biocompatibility of PDMS specimens after the three processes was not significantly different from that of pure PDMS (OD: 0.22–0.23). In addition, FDM 3D printing leads to poor PDMS translucency (transparency < 0.5) when flipping the mold due to low printing accuracy (100 μm), and although some studies have demonstrated that using organic solvent vapor treatment of the mold will improve it, the resulting flipping accuracy is difficult to control, while the HTL mold is more accurate (10 μm) and its flipped PDMS fabricated light transmission (0.96) is almost no worse than pure PDMS. Further, in terms of mechanical properties, the ultimate elongation of the PDMS specimens made using the sacrificial template method was measured to be greater than that of the PDMS made using the HTL mold flip due to the effect of organic solvent on its swelling. In this paper, the effect of different hollow occupancy ratios of flexible hollow telescopic rods, in both radial and axial directions, on their ultimate elongation was investigated. The results show that the ultimate elongation of the telescopic rod structure increases gradually with the increase of the hollow percentage for the telescopic experiments. At the same time, the telescopic experiments show that for the same elongation, the larger the hollow percentage, the smaller the required thrust force. Although this paper focused on comparing manufacturing processes for hollow telescopic rod structures, it has important implications for researchers working in related fields. The research ideas in this paper can serve as a valuable reference point for those seeking to fabricate other types of equipment structures and can provide a corresponding process comparison reference when performing fabrication of other types of structures.

## Data Availability

Not applicable.

## Supplementary Materials

The following supporting information can be downloaded at: https://www.mdpi.com/article/10.3390/mi14061105/s1, Figure S1: (a–c) respectively representing PLA-mold, ABS-mold, HTL-mold; Figure S2: Experimental load–displacement curves of telescopic rod of different heights of hollow rod (a) H_h_ = 0 mm, R_h_ = 0.5 mm, (b) H_h_ = 3.75 mm, R_h_ = 0.5 mm. (c) H_h_ = 7.5 mm, R_h_ = 0.5 mm (d) H_h_ = 11.25 mm, R_h_ = 0.5 mm. (e) H_h_ = 15 mm, R_h_ = 0.5 mm (f) H_h_ = 7.5 mm, R_h_ = 0.6 mm (g) H_h_ = 7.5 mm, R_h_ = 0.7 mm (h) H_h_ = 7.5 mm, R_h_ = 0.8 mm; Figure S3: (a) Comparison of experimental load–displacement curves of hollow telescopic rods of different radii (b) Comparison of experimental load–displacement curves of hollow telescopic rods of different heights (c) Comparison of maximum stretch-displacement of specimens produced via various turn-flipped-molding processes.; Figure S4: Strain of FEA-cloud-map; Table S1: Mechanical parameters of PDMS; Table S2: The test-bar size difference between design and fabrication; Table S3: Material models of PDMS and their corresponding material constants obtained from experimental data.
