# Supplementary material for: Research on the Comparison Properties of PDMS Specimens Demolding Processes and the Mechanical Performance of Hollow-Solid Ratios of Flexible Telescopic Rods
Source: Micromachines (Basel). 2023 May 24;14(6):1105. doi: 10.3390/mi14061105 (PMC10304836; doi:10.3390/mi14061105)
Supplement: Supplementary file 1 [file micromachines-14-01105-s001.zip › micromachines-2416342-supplementary.pdf]

## Supporting Information

# Research on the comparison properties of PDMS Specimens Demolding Processes and the mechanical Performance of Hollow-Solid Ratios of flexible telescopic rods

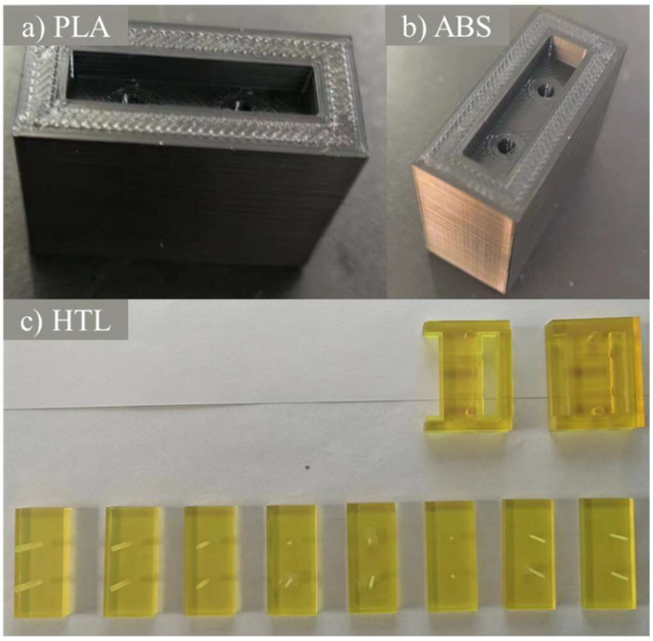


Figure S1. (a, b, c) respectively representing PLA-mold, ABS-mold, HTL-mold.

Table S1. Mechanical parameters of PDMS.

| Material | Density (kg/m$^3$) | Poisson's ratio | Young's modulus (MPa) |
|---|---|---|---|
| Sylgard 184 | 0.97 | 0.49 | 1.47 |

Table S2. The test-bar size difference between design and fabrication.

| Exp.no. | Sample hollow rod height: (mm) | | | | Sample hollow rod radius: (mm) | | | | Mold-material |
|---|---|---|---|---|---|---|---|---|---|
| | Designed | Molding | Difference | %Increase/decrease | Designed | Molding | Difference | %Increase/decrease | |
| 1 | 0 | 0 | 0 | 0 | 0.5 | 0.49 | 0.01 | -2% | HTL |
| 2 | 3.75 | 3.74 | 0.01 | -0.27% | 0.5 | 0.49 | 0.01 | -2% | HTL |
| 3 | 7.5 | 7.49 | 0.01 | -0.13% | 0.5 | 0.49 | 0.01 | -2% | HTL |
| 4 | 11.25 | 11.23 | 0.02 | -0.18% | 0.5 | 0.5 | 0 | 0 | HTL |
| 5 | 15 | 14.97 | 0.03 | -0.2% | 0.5 | 0.49 | 0.01 | -2% | HTL |
| 6 | 7.5 | 7.48 | 0.02 | -0.27% | 0.6 | 0.59 | 0.01 | -1.67% | HTL |
| 7 | 7.5 | 7.49 | 0.01 | -0.13% | 0.7 | 0.68 | 0.02 | -2.86% | HTL |
| 8 | 7.5 | 7.51 | 0.01 | +0.13% | 0.8 | 0.78 | 0.02 | -2.5% | HTL |
| 9 | 7.5 | 7.61 | 0.11 | +1.46% | 0.5 | 0.55 | 0.05 | 10% | ABS |
| 10 | 7.5 | 7.63 | 0.13 | +1.72% | 0.5 | 0.57 | 0.07 | 14% | PLA |

Table S3. Material models of PDMS and their corresponding material constants obtained from experimental data.

| Material model | Material constants | PDMS-AB(base polymer:curing agent) |
|---|---|---|
| Mooney-Rivlin | C01(MPa) | -1.6047 |
| | C10(MPa) | 2.0019 |
| Neo-Hooken | Initial shear Modulus μ(MPa) | 1.1994 |
| | Incompressibility parameter $D_1$ | 0 |
| Yeoh | Incompressibility parameter $D_1$ | 0 |
| | C10(MPa) | 0.59968 |
| Odgen | Incompressibility parameter $D_1$ | 0 |
| | α | 6.0593 |
| | μ(MPa) | 0.24937 |

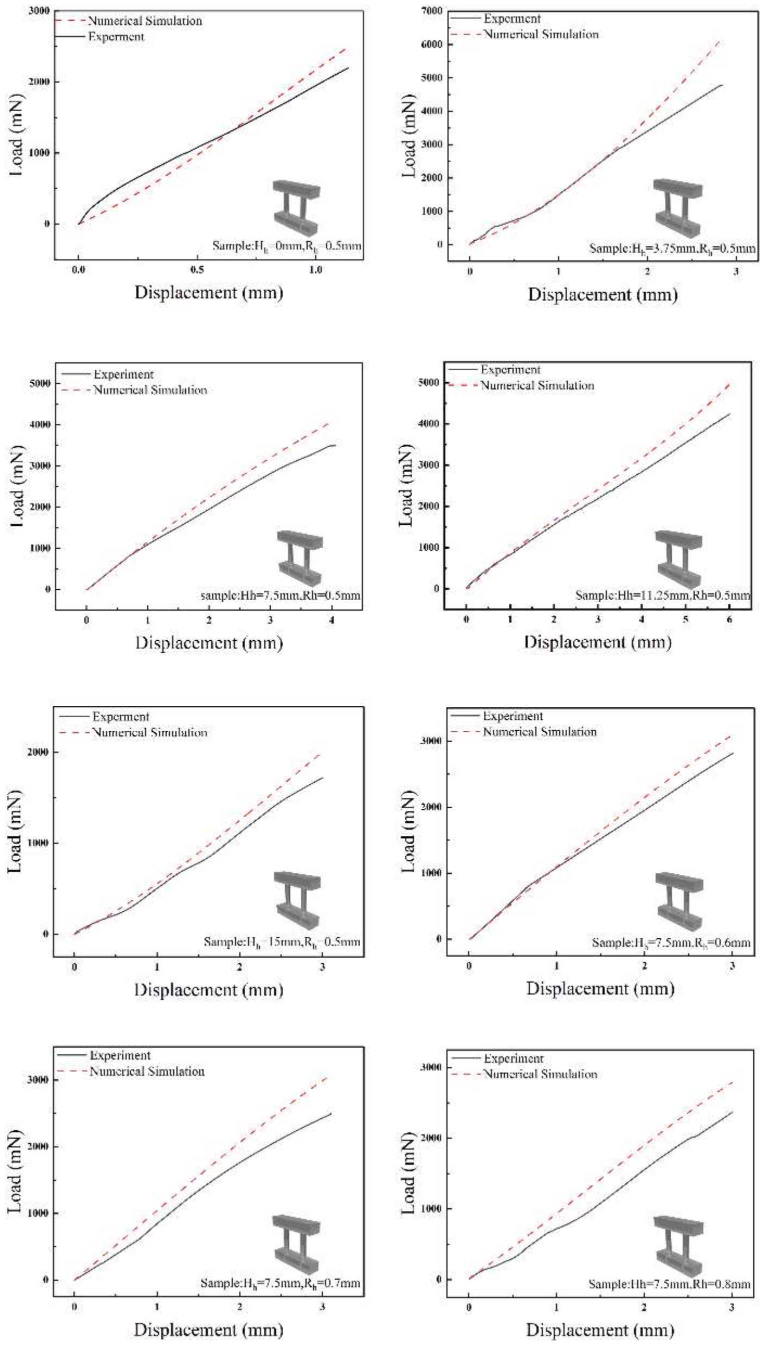


Figure S2. Experimental load-displacement curves of telescopic rod of different heights of hollow rod.

(a) $H_h = 0$ mm, $R_h = 0.5$ mm, (b) $H_h = 3.75$ mm, $R_h = 0.5$ mm. (c) $H_h = 7.5$ mm, $R_h = 0.5$ mm. (d) $H_h = 11.25$ mm, $R_h = 0.5$ mm. (e) $H_h = 15$ mm, $R_h = 0.5$ mm. (f) $H_h = 7.5$ mm, $R_h = 0.6$ mm. (g) $H_h = 7.5$ mm, $R_h = 0.7$ mm. (h) $H_h = 7.5$ mm, $R_h = 0.8$ mm.

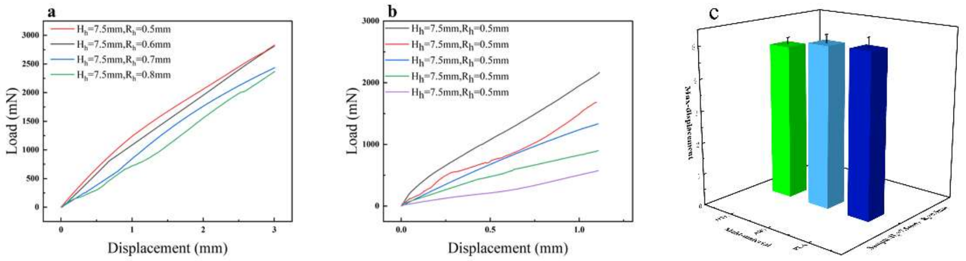


Figure S3. (a) Comparison of experimental load-displacement curves of hollow telescopic rods of different radii.

(b) Comparison of experimental load-displacement curves of hollow telescopic rods of different heights.

(c) Comparison of maximum stretch-displacement of specimens produced via various turn-flipped-molding processes.

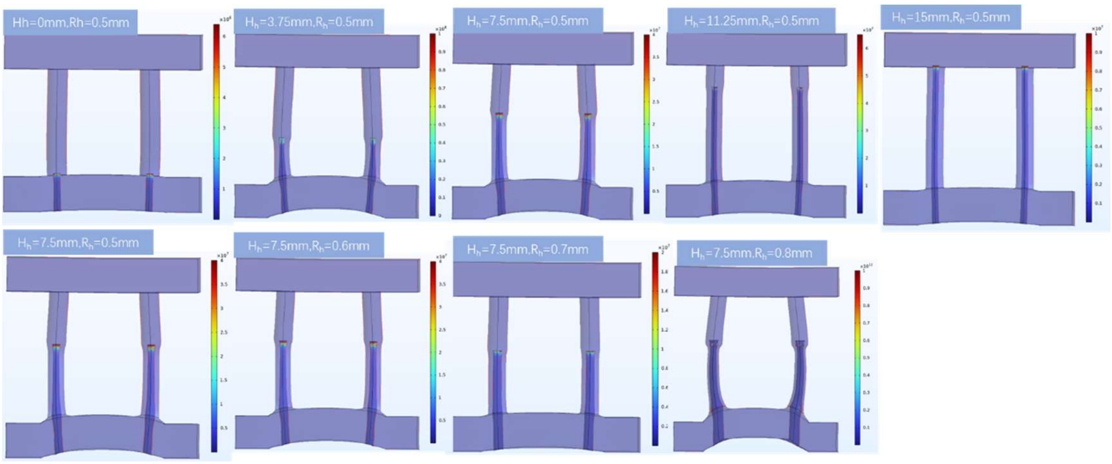


Figure S4. strain of FEA-cloud-map.
